# Measuring Digital Vaccine Literacy: Development and Psychometric Assessment of the Digital Vaccine Literacy Scale

**DOI:** 10.2196/39220

**Published:** 2022-12-14

**Authors:** Ilaria Montagni, Aude Pouymayou, Edwige Pereira, Christophe Tzourio, Stéphane Schück, Nathalie Texier, Juan Luis González-Caballero, The CONFINS Group

**Affiliations:** 1 Bordeaux Population Health UMRS1219 University of Bordeaux Institut national de la santé et de la recherche médicale Bordeaux France; 2 Kappa Santé Paris France; 3 Kap Code Paris France; 4 Department of Statistics and Operational Research University of Cádiz Cádiz Spain; 5 See Acknowledgments

**Keywords:** Internet, literacy, measurement, vaccination, vaccine, health information, health literacy, online, content, validity, reliability, digital literacy

## Abstract

**Background:**

The use of the internet to look for information about vaccines has skyrocketed in the last years, especially with the COVID-19 pandemic. Digital vaccine literacy (DVL) refers to understanding, trust, appraisal, and application of vaccine-related information online.

**Objective:**

This study aims to develop a tool measuring DVL and assess its psychometric properties.

**Methods:**

A 7-item online questionnaire was administered to 848 French adults. Different psychometric analyses were performed, including descriptive statistics, exploratory factor analysis, confirmatory factor analysis, and convergent and discriminant validity.

**Results:**

We developed the 7-item DVL scale composed of 3 factors (understanding and trust official information; understanding and trust information in social media; and appraisal of vaccine information online in terms of evaluation of the information and its application for decision making). The mean DVL score of the baseline sample of 848 participants was 19.5 (SD 2.8) with a range of 7-28. The median score was 20. Scores were significantly different by gender (*P*=.24), age (*P*=.03), studying or working in the field of health (*P*=.01), and receiving regular seasonal flu shots (*P*=.01).

**Conclusions:**

The DVL tool showed good psychometric proprieties, resulting in a promising measure of DVL.

## Introduction

Vaccination is one of the most commonly queried topics on the internet [1]. With the COVID-19 pandemic, the number of people seeking vaccine-related information on the internet has skyrocketed [2,3]. The Increasing Vaccination Model [4] states that information sharing and rumors contribute, among other factors, to motivation to vaccinate. The 5C (complacency, constraints, calculation, confidence, collective responsibility) Model [5] asserts that vaccine hesitancy depends also on the engagement in extensive information seeking (ie, calculation), which determines deliberation on the risks and benefits of vaccination based on retrieved data and news. Thus, according to these 2 models, the contents of online information have the potential to determine the decision to get vaccinated or not.

Online sources for vaccine-related information vary. These include websites of official institutions, blogs, forums, social media, among others. The information they convey can be either reliable and valid or unscientific and misleading. On the one hand, social media have been defined as a powerful catalyst for the “anti-vax movement” [6]. This has been emphasized during the COVID-19 pandemic with a wide circulation of false information about vaccines on social media platforms [7,8]. On the other hand, websites of official institutions, such as those of governments, are considered to be more accurate [9]. Recent studies concerning the COVID-19 pandemic have confirmed that government websites are the most trusted source of information [10,11].

Hesitancy toward vaccination remains a present and growing issue [12]. Among the various reasons for this attitude, *misconception* and *misinformation* can have a strong impact [13]. Online messages can contribute to diffuse controversial information and induce indecision and skepticism about vaccines [14].

Preliminary studies have explored the influence of the internet on growing vaccine hesitancy [15,16]. According to these studies, those who search for online information more actively are usually also the most hesitant, trusting and believing science less than other sources [17]. Furthermore, the spread of fake news and misinformation on social media is blamed as a primary cause of vaccine hesitancy [18]. However, the internet is also a source of official reliable information and might provide new instruments to fight against vaccine hesitancy, because users can also access government websites, for instance.

Digital health literacy refers to the capacity of people to adequately understand and process online health information to meet their needs [19]. This set of skills affects the health of users, as well as the quality of their health care, orienting their health behavior. Vaccine literacy is defined as not only knowledge about vaccines, but also developing a simple system to communicate and offer vaccines as a sine qua non of a functioning health system [20,21]. Digital vaccine literacy (DVL) is a construct mixing digital health literacy and vaccine literacy. DVL theoretically affects both motivation and skills involving online information seeking for clear-cut elucidated decision making about getting vaccinated or not.

A valid tool for measurement of DVL is thus essential to provide inputs to train people in better navigating vaccine-related information on the internet on both social media and official online sources. This scale developed herein also allows to provide a general and population-based assessment of DVL: given the spread of the COVID-19 pandemic and the relevance of accepting vaccination, today more than ever it is pivotal to investigate the level of DVL in the population and examine its potential contribution to vaccine uptake. Furthermore, the scale can be used as an instrument to measure the effectiveness of interventions aimed at increasing DVL for reducing vaccine hesitancy.

To the best of our knowledge, no tool exists to measure DVL. The currently used questionnaires focus on vaccine literacy in general and not on online vaccine literacy (ie, DVL) [21,22]. The aim of this study was to describe the development and psychometric properties of a scale measuring DVL (Multimedia Appendix 1).

## Methods

### Overview of Study Phases

Our study was conducted in 3 distinct phases: (1) development of a tool to measure DVL, (2) collection of empiric cross-sectional data from a French adult population sample, and (3) assessment of the psychometric properties of the DVL tool.

We used the COSMIN (Consensus-Based Standards for the Selection of Health Measurement Instruments) to develop the DVL tool and validate it [23].

### Phase 1: DVL Tool Development

We based the conception of the DVL tool on the theories of digital health literacy and vaccine literacy, investigating the understanding, trust, appraisal, and application of vaccine-related information online [20,24], with the distinction between social media/forums and government websites. A panel of 5 public health researchers proposed a series of items inspired by the Health Literacy Questionnaire [25,26], the eHealth Literacy Scale [19], and the Vaccine Literacy Scale [22].

The construct of DVL was decided a priori and defined before any item activity. Expert judges confirmed through literature review that there were no existing instruments that will adequately serve the same purpose. A deductive method was used to identify the items through the description of the relevant field (domain), in combination with an inductive method based on the exchanges among experts. A group of 10 volunteers with characteristics similar to the target population pretested the questions. Items were worded in simple terms and unambiguously.

We narrowed the items focusing on vaccination and the digital environment to eventually obtain a total of 7 questions answered on a 4-point Likert scale (from 4 [agree] to 1 [disagree]) and an additional answer option “I do not know, I do not look for vaccine-related information.” This latter option was taken into account in the descriptions, but was considered “noninformative” for the analysis of the structural validity of the scale. The total score of the DVL scale was calculated through the sum of all answers to the items. The score of the scale varied from 7 to 28. The higher the score, the better the DVL level.

We also included an item on “the online sources which were the most consulted for vaccine-related information seeking” (online journals, government websites, health institution websites, social media, forums, video platforms, other). Finally, participants had to rate the importance of the use of the internet for vaccine-related information seeking through a visual analog scale from 1 (not important at all) to 5 (very important).

### Phase 2: Data Collection and Definition of the Population Under Study

We administered the DVL tool to participants from an open online cohort (CONFINS) [27]. All participants were aged more than 18 years, living in France, and were able to read and understand French. CONFINS is a cohort collecting data on the impact of confinement on the health and well-being of the French population [28]. It included, among others, variables on opinions about vaccination and the DVL items. It also comprised sociodemographic information (age, gender, having children, being vaccinated against influenza) used in this study. Items were defined by a group of public health experts through several rounds of corrections and refinement. CONFINS consisted in a baseline questionnaire and repeated monthly follow-up questionnaires. Participants could decide whether to be contacted or not for the following phases of the survey. This study used data from the baseline questionnaire and the first follow-up questionnaire, covering the period from April to May 2020. This was a convenience sample.

CONFINS participants were recruited on a voluntary basis with no incentives through different communication channels. Posts were published on the social media (LinkedIn, Twitter, Facebook) of the University of Bordeaux and the partner contract research organization hosting the database. A total of 3 press releases were addressed to journalists. The coprinciple investigators were interviewed to promote the study. Three newsletters and weekly emails and SMS text messages were sent to the participants to remind them to complete the follow-up questionnaires. All recruitment strategies directed potential participants toward the CONFINS website including information on the objectives of the study and the investigators. Informed consent, containing details on the length of time of the survey, stored data, investigators and objectives of the study, was provided through an electronic signature.

### Study Population

Concerning the population of this study, we included all participants completing all items of the DVL tool, comprising also those choosing the answer option “I do not know, I do not look for vaccine-related information” (N=2935). However, for the sake of the specific analyses required to evaluate the psychometric properties of the DVL tool, we obtained a subsample of 848 participants who did not use the answer option “I do not know, I do not look for vaccine-related information.” The choice of using mainly the subsample was justified by the fact that the factor analysis mentioned later requires ordering the response modalities. As the “I do not know, I do not look for vaccine-related information” modality is difficult to classify, we decided to remove it. The subsample included those who had completed the baseline questionnaire (“test” phase). Among them, 62 participants also answered the follow-up questionnaire (“retest” phase).

### Phase 3: Analysis of Other Psychometric Properties of the DVL Tool

First, a descriptive analysis of each item of the scale was performed for both the total sample of participants (N=2935) and the subsample (n=848). Participants of the subsample were also described according to their sociodemographic characteristics (ie, age, gender, working/studying in the field of health, having children, and being regularly vaccinated against flu). For quantitative variables, the mean and SD were calculated. For qualitative variables, participants were described in numbers and percentages. Answers to items were compared for each aforementioned sociodemographic characteristic. To do this, the item response options were grouped into “agree”/“rather agree” versus “disagree”/“rather disagree.” The statistical tests of *χ*^2^ independence were used to compare the responses of the participants according to their sociodemographic criteria.

Second, an exploratory factor analysis (EFA) was performed on the baseline data to identify the underlying latent factors in the set of items as well as their association. As the items were ordinal variables, the polychoric correlation matrix of observed items was explored. Two initial hypotheses were tested. The first was the test of Bartlett sphericity. If the test was significant (*P*<.05), the observed matrix was significantly divergent from the null matrix and an EFA had to be performed. The second hypothesis required testing the measure of sampling adequacy using the Kaiser-Meyer-Olkin index [29]. This is a measure of the proportion of variance among the observed items, equivalent to the common variance. Thus, it was used to verify for partial correlations. If the Kaiser-Meyer-Olkin index was above 0.50, the EFA was adequate. Next, the number of factors to be kept in the model had to be chosen based on different criteria using eigenvalues. The Kaiser criterion consisted of keeping factors with eigenvalues greater than 1. The Cattell criterion (also called the “elbow criterion”) was based on identifying the inflection point, where the slope of the eigenvalue curve according to the number of factors in the model stabilized well below the “elbow.” Thus, the number of factors above the point was retained. The third criterion was the use of a parallel analysis. In this analysis, the eigenvalues obtained were compared with those that would be obtained from random data. The number of factors extracted was the number of factors whose eigenvalues were higher than those found with random data. In addition, the item × factor matrix had to be rotated to better identify how the items were substantially related to each factor. Among the several approaches to rotation, the oblique rotation was used because it considers the correlation between factors [30]. Finally, the items were associated with a factor when their saturation weight was close or superior to 0.30 and their communalities were considered as acceptable above 0.20. We also performed a confirmatory factor analysis (CFA) considering the criteria root-mean-square error of approximation (acceptable range between 0.08 and 0.1), comparative fit index (acceptable range >0.90) and standardized root-mean-square error (acceptable range between 0 and 0.008).

Third, to complete the validation of the DVL scale, the convergent and discriminant validities of the score were assessed. The sociodemographic criteria of participants with a low DVL score were compared with those of participants with a high score, determined according to the median, using *χ*^2^ statistical tests of independence.

Statistical significance was considered if *P*<.05 and all tests were 2-tailed. Statistical analyses were performed on SAS version 9.3 software (SAS Institute).

### Ethics Approval

The study was approved by the French Committee for the Protection of Individuals (Comité de Protection des Personnes [CPP], approval number 46-2020) and the French National Agency for Data Protection (Commission Nationale de l'Informatique et des Libertés [CNIL], approval number MLD/MFI/AR205600). The study follows the principles of the Declaration of Helsinki and the collection, storage, and analysis of the data comply with the European Union General Data Protection Regulation (EU GDPR).

## Results

### Descriptive Analysis

Responses to the 7 items on the DVL tool by the total sample and the subsample are reported in Tables 1 and 2, respectively.

**Table 1:** Results of all potentials items of the DVL scale^a^ in the CONFINS online cohort (N=2935).

Items	Disagree, n (%)	Rather disagree, n (%)	Rather agree, n (%)	Agree, n (%)	Do not know, n (%)
1. I find vaccine-related information on social media and forums is understandable	215 (7.33)	478 (16.29)	582 (19.83)	134 (4.57)	1526 (51.99)
2. I find vaccine-related information on government websites is understandable	111 (3.78)	176 (6)	1394 (47.50)	586 (19.97)	668 (22.76)
3. I can detect vaccine-related fake news	97 (3.30)	477 (16.25)	1500 (51.11)	821 (27.97)	40 (1.36)
4. I trust vaccine-related information provided by government websites	55 (1.87)	191 (6.51)	1250 (42.59)	948 (32.30)	491 (16.73)
5. I find vaccine-related information on social networks is valid	533 (18.16)	1123 (38.26)	134 (4.53)	26 (0.89)	1119 (38.13)
6. When I read vaccination information online, I cross-reference it with other sources to verify its validity	178 (6.06)	394 (13.42)	1288 (43.88)	1060 (36.12)	15 (0.51)
7. I think the information I find online may influence my decision to get vaccinated	413 (14.07)	649 (22.11)	918 (31.28)	231 (7.97)	724 (24.67)

^a^DVL scale: Digital Vaccine Literacy scale.

**Table 2:** Results of all potential items of the DVL scale^a^ in the CONFINS online cohort (n=848, without “do not know”).

Item	Disagree, n (%)	Rather disagree, n (%)	Rather agree, n (%)	Agree, n (%)	Test-retest reliability (n=62), intraclass correlation coefficient (95% CI)
1. I find vaccine-related information on social media and forums is understandable	139 (16.4)	287 (33.8)	342 (40.3)	80 (9.4)	0.14 (0.01 to 0.37)
2. I find vaccine-related information on government websites is understandable	49 (5.8)	82 (9.7)	492 (58.0)	225 (26.5)	0.53 (0.33 to 0.69)
3. I can detect vaccine-related *fake news*	27 (3.2)	111 (13.1)	421 (49.6)	289 (34.1)	0.70 (0.55 to 0.81)
4. I trust vaccine-related information provided by government websites	23 (2.7)	82 (9.7)	409 (48.2)	334 (39.4)	0.46 (0.24 to 0.63)
5. I find vaccine-related information on social networks is valid	224 (26.4)	529 (62.4)	83 (9.8)	12 (1.4)	0.05 (0.01 to 0.29)
6. When I read vaccination information online, I cross-reference it with other sources to verify its validity	44 (5.2)	87 (10.3)	365 (43)	352 (41.5)	0.48 (0.27 to 0.65)
7. I think the information I find online may influence my decision to get vaccinated	122 (14.4)	267 (31.5)	354 (41.7)	105 (12.4)	–0.09 (–0.33 to 0.16)

^a^DVL scale: Digital Vaccine Literacy scale.

The “I do not know, I do not look for vaccine-related information” response rates were 51.99% (1526/2935) for item 1, 22.76% (668/2935) for item 2, 1.36% (40/2935) for item 3, 16.73% (491/2935) for item 4, 38.13% (1119/2935) for item 5, 5.04% (148/2935) for item 6, and 24.67% (724/2935) for item 7. Per participant, the maximum number of “I do not know, I do not look for vaccine-related information” was 5; 24.74% (726/2935) responded “I do not know, I do not look for vaccine-related information” for at least one item; 23.51% (690/2395) for at least two items; 10.97% (322/2935) for at least three items; 7.97% (234/2935) for at least four items; and 3.92% (115/2395) for at least five items. The mean of responses per participant was 1.56 (SD 1.4). In addition, the use of a factor analysis requires ordering the response modalities. As the “I do not know, I do not look for vaccine-related information” modality is difficult to classify in view of the others, we decided to remove it from the analyses. Therefore, the study sample contained 848 participants who responded to the items as shown in Table 2.

All item response options were used, thus qualifying them as informative. In addition, Table 2 shows that the items were discriminating because the response rates for each modality were in the average. The intraclass correlation coefficient (ICC) was calculated based on data from the 62 participants. Items 1, 5, and 7 presented a low ICC, which could be explained by nonconcordant responses between the 2 measurements, and therefore less reliability, their formulation, and possible difficulty in answering them. In fact, these items had the highest percentages of the “I do not know, I do not look for vaccine-related information” responses (Table 1).

In the subsample of 848 participants, 73.1% (620/848) were females. The mean age was 29.9 (SD 12.3). Participants working or studying in the field of health were 397/848 (46.8%). The percentage of parents was 20.9% (178/848) and 557/848 (65.7%) were not vaccinated against flu (Table 3).

The mean of the importance of the use of the internet for vaccine-related information seeking was 3.7 out of 5 (SD 1.1). The most used source for vaccine-related information seeking was websites of health institutions (395/848, 46.6%), followed by government websites (184/848, 21.7%). Online journals were consulted by 56/848 individuals (6.6%), whereas other sources by 37/848 individuals (4.4%). Social networks were consulted by 70/848 individuals (8.3%), video platforms by 16/848 (1.9%), and forums by 8/848 (0.9%).

Multimedia Appendix 2 reports data on the comparison of the answer to the DVL items according to sociodemographic characteristics.

Regarding their answers to the items, women were more in agreement with the statement of item 3 (I can detect vaccine-related fake news), item 4 (I trust vaccine-related information provided by government websites), and item 7 (I think the information I find online may influence my decision to get vaccinated) than men. Participants aged 35 or over disagreed with item 1 (I find vaccine-related information on social media and forums is understandable), which was different from those under 35 years. Participants studying or working in the field of health and those receiving regular flu shots were more in agreement with items 2 (I find vaccine-related information on government websites is understandable), item 3 (I can detect vaccine-related fake news), and item 4 (I trust vaccine-related information provided by government websites) and disagreed with item 7 (I think the information I find online may influence my decision to get vaccinated) compared with those who worked or studied in another field and those who did not get a flu shot. There was no difference in responses concerning parenthood.

**Table 3:** Sociodemographic characteristics of the CONFINS study population.

Characteristics		Value
Age, mean (SD)		29.9 (12.3)
**Categories** **(n=835),** **years** **, n (%)**		
	18-34	653 (78.2)
	≥35	182 (21.8)
**Gender (n=848), n (%)**		
	Female	620 (73.1)
	Male	228 (26.9)
**Study or work in the** **field of health** **(n=763), n (%)**		
	No	366 (48.0)
	Yes	397 (52.0)
**Children (n=848), n (%)**		
	No	670 (79.0)
	Yes	178 (21.0)
**Influenza vaccine (n=848), n (%)**		
	No	557 (65.7)
	Yes	291 (34.3)

### Exploratory Factor Analysis

The interitem polychoric correlation matrix was used for the first definition of the associations between items (Table 4).

In the polychoric matrix, we observed strong correlations between items 2, 3, and 4. Item 1 was more correlated with item 5.

The hypotheses justifying the performance of an EFA were validated. The Bartlett test of sphericity showed a *P*<.05 (*χ*^2^_21_=1319.37) and the Kaiser-Meyer-Olkin index was 0.58, indicating good sampling adequacy.

**Figure 1:**
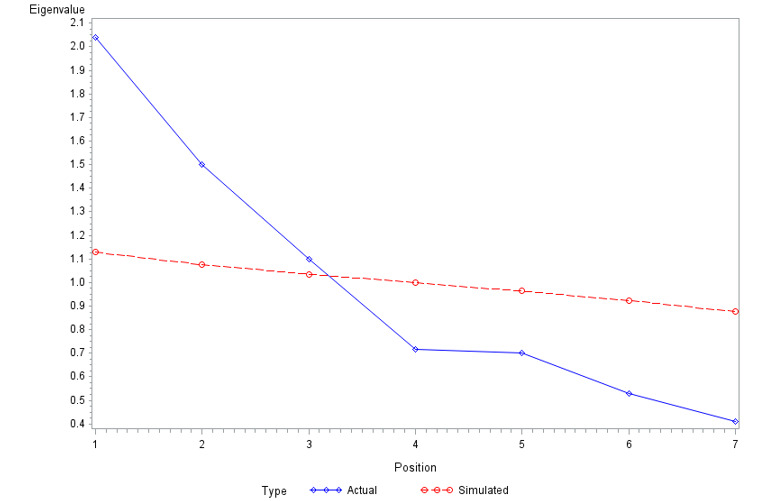
Distribution of the median simulated eigenvalues according to the number of factors and application of the parallel analysis. 7 variables, iterations, 848 observations.

The number of factors was calculated based on the Kaiser and Cattell criteria and the parallel analysis; 3 factors were kept (Figure 1).

Finally, several EFAs were performed to test the different oblique rotations. The OBLIMIN oblique rotation was the most common. Table 5 shows that items 1 and 5 were associated with factor 2; items 2, 3, and 4 with factor 1; and items 6 and 7 with factor 3. The oblique rotation OBEAQUAMAX showed that saturation weights revealed several possible associations between items and factors. Items 3 and 7 were associated with both factors 1 and 3 based on the saturation weights close or superior to 0.30. Communalities were all acceptable.

**Table 4:** Interitem polychoric correlation matrix.

Item	1	2	3	4	5	6	7
1	—^a^	—	—	—	—	—	—
2	0.33	—	—	—	—	—	—
3	0.00	0.46	—	—	—	—	—
4	0.06	0.64	0.52	—	—	—	—
5	0.45	–0.02	–0.10	–0.06	—	—	—
6	0.06	0.19	0.34	0.12	–0.02	—	—
7	0.13	–0.11	–0.13	–0.15	0.21	0.20	—

^a^Dashes correspond to the absence of a correlation between items.

**Table 5:** Matrices of the saturation weights with oblique rotations and item communalities.

Item	OBLIMIN			OBEAQUAMAX			Communality
	Factor 1	Factor 2	Factor 3	Factor 1	Factor 2	Factor 3	
1	0.19	0.69	–0.02	0.19	0.67	0.01	0.46
2	0.78	0.23	–0.01	0.74	0.21	0.13	0.63
3	0.60	–0.14	0.25	0.50	–0.15	0.37	0.47
4	0.76	0.01	–0.03	0.72	–0.01	0.12	0.57
5	–0.08	0.56	0.03	–0.07	0.57	–0.01	0.34
6	0.17	–0.05	0.49	0.03	–0.04	0.53	0.28
7	–0.23	0.20	0.33	-0.30	0.21	0.29	0.21

Table 6 shows the interfactor correlations according to the OBLIMIN and OBEAQUAMAX rotations. Correlations were low but factor 1 was negatively correlated with factor 2, and factor 3 was positively correlated with the other 2 factors.

In view of these results, the relationships between the items and the factors were interpreted as follows. Factor 1 was associated with items relating to “reliable” information about vaccination (government sites), with the label “understanding and trust official information about vaccination provided by institutional websites.” Factor 2 was associated with items related to information about vaccination of which 1 should be relatively “unreliable” (social media) with the label “understanding and trust information about vaccines as provided by social media.” Finally, factor 3 was associated with items related to the application of knowledge on vaccination consulted on the web (label of factor 3).

Finally, we also performed a CFA to confirm these 3 dimensions (Table 7).

In the CFA the criterion values were as follows: root-mean-square error of approximation 0.12 (90% CI 0.11-1.14), comparative fit index 0.80, and standardized root-mean-square error 0.08.

**Table 6:** Interfactor correlation matrices (OBLIMIN and OBEAQUAMAX).

Factor	OBLIMIN			OBEAQUAMAX		
	Factor 1	Factor 2	Factor 3	Factor 1	Factor 2	Factor 3
1	1	—^a^	—	1	—	—
2	–0.08	1	—	–0.09	1	—
3	0.11	0.18	1	0.19	0.16	1

^a^Dashes correspond to the absence of a correlation between items and factors.

**Table 7:** Weights of the relationships item-factors of the final model by confirmatory factor analysis.

Item	Model 1		
	Factor 1	Factor 2	Factor 3
1	—^a^	0.87	—
2	0.56	—	—
3	0.43	—	—
4	0.51	—	—
5	—	0.23	—
6	—	—	0.83
7	—	—	0.15

^a^Dashes correspond to the absence of a correlation between items and factors.

### Convergent and Discriminant Validity

The mean DVL score of the baseline sample of 848 participants was 19.5 (SD 2.8). Participants scored between 14 and 21 points (ie, in the medium DVL range). The median was 20.

Table 8 shows the sociodemographic characteristics of the sample according to the DVL level. The score was dichotomized into <20 (low DVL score) and ≥20 (high DVL score).

Participants with a low DVL level were significantly older (30.8 years vs 29 years; *P*=.03). Those working or studying in the field of health were significantly more numerous in the group with a higher score (*P*=.01). Those who did not receive regular flu vaccinations were significantly more likely to be in the low score group (*P*=.01). Among online sources for vaccine-related information, government websites were more used by those with a higher DVL (*P*=.03). Those with a score less than 20 considered the use of the internet for vaccine-related information less important than others, with the means being 3.4 (SD 1.1) and 4.0 (0.9), respectively.

**Table 8:** Sociodemographic characteristics of the baseline sample by DVL^a^ level (n=848).^b^

Sociodemographics			Low DVL (score <20)		High DVL (score ≥20)		*P* value
Age (years), mean (SD)			30.8 (12.9)		29.0 (11.7)		.03
**Age c** **ategories (n=397)**							.04
	18-34	298/397 (75.1)		355/438 (81.1)			
	≥35	99/397 (24.9)		83/438 (18.9)			
**Gender (n=404)**							.24
	Female	303/404 (75)		317/444 (71.4)			
	Male	101/404 (25)		127/444 (28.6)			
**Studying or working in the** **field of health** **(n=357)**							.01
	No	192/357 (53.8)		174/406 (42.9)			
	Yes	165/357 (46.2)		232/406 (57.1)			
**Having children** **(n=404)**							.38
	No	314/404 (77.7)		356/444 (80.2)			
	Yes	90/404 (22.3)		88/444 (19.8)			
**Vaccinated against flu** **(n=404)**							.01
	No	283/404 (70)		274/444 (61.7)			
	Yes	121/404 (30)		170/444 (38.3)			
**Online sources for vaccine-related information** **(n=338)**							.03
	Online journals	30/338 (8.9)		26/390 (6.7)			
	Government websites	73/338 (21.6)		111/390 (28.5)			
	Health institutions websites	185/338 (54.7)		210/390 (53.8)			
	Social media	19/338 (5.6)		13/390 (3.3)			
	Forums	7/338 (2.1)		1/390 (0.3)			
	Video Platforms	5/338 (1.5)		11/390 (2.8)			
	Other	19/338 (5.6)		18/390 (4.6)			
Importance of the use of the internet for vaccine-related information seeking (n=338), mean (SD)			3.4 (1.1)^c^		4.0 (0.9)^d^		<.001

^a^DVL: digital vaccine literacy.

^b^Values are presented as n/N (%) unless indicated otherwise.

^c^N=338.

^d^N=390.

## Discussion

### The DVL Scale: Dimensions, Items, and Answer Options

We conceived a scale measuring DVL and assessed its psychometric proprieties among a sample of French adults. The scale was composed of 7 items covering the overarching construct of DVL, which includes 3 subdimensions. The first subdimension (items 2 and 4) refers to understanding and trusting official information about vaccination provided by institutional websites. The second subdimension (items 1 and 5) refers to understanding and trusting information about vaccines as provided by social media. The underlying assumption for these 2 dimensions is that government websites provide valid information while social media provide fake news [31]. In this line, in our sample, the most accessed sources were health institutions and government websites, while social media and forums were less consulted.

The third subdimension (items 3, 6, and 7) refers to the appraisal of vaccine information online in terms of evaluation of the information and its application for decision making. Two items (3 and 7) are actually included in both subdimensions 1 and 2. For the item “I can detect fake news,” this ambivalence can be explained by the fact that recognizing fake news is a reflection of both the understanding/trust of official information (subdimension 1) and the appraisal and practical application of found information (subdimension 3). The possible explanation is that those who recognize fake news are more inclined to government websites and are more cautious in interpreting vaccine-related information. The inclusion of the item “I think the information I find online may influence my decision to get vaccinated” in both subdimensions 1 and 3 can be interpreted as the fact that trusting official information might correspond to a higher capacity to make correct evidence-based decisions about vaccination. This overlap of factors infers an interrelation of items, which can suggest that the scale is coherent and congruent.

Some recommendations must be considered when using the DVL scale. There are 4 response options (*disagree*, *rather disagree*, *rather agree*, and *agree*) that are used to obtain a score. However, even if it does not contribute to the calculation of the score, the fifth response option (I do not know, I do not look for vaccine-related information) provides useful information. First, this option respects the opinion of those not feeling concerned without forcing or biasing their answer. Second, it is really interesting to measure the percentage of those who do not feel concerned by seeking vaccine-related information online. In this study, one-half of the participants used the option “I do not know, I do not look for vaccine-related information” for the item on understanding information found on social media, and more than one-third for the item on trust in social media. These results confirm the fact that social media are more rarely used than government websites for this type of information. Thus, we suggest to calculate the score by considering as missing values all cases including 1 response option “I do not know, I do not look for vaccine-related information”, and to complete this information with the percentage of those using this same option. These data are complementary in measuring DVL.

### The DVL Scores of the Study Sample

Having a low DVL score (<20) can be interpreted as a relevant alarm in relation to the extensive use of the internet for vaccine-related contents, especially in France [15]. As is the case with health literacy, low DVL scores are associated with a higher risk of adopting an unhealthy behavior [32]; in this case this refers to the decision of *not to get vaccinated*. Not being able to navigate information on the internet could increase the chance of having a negative perception about vaccines [33]. Lower scores in the scale would also correspond to the incapacity to recognize fake news and trust in unofficial information provided by social media. There are many who consult the internet regarding vaccination and it is important to know their levels of DVL to help them navigate online information.

DVL scores were significantly different by age (participants with a low DVL score were significantly older), studying or working in the field of health (those working or studying in the field of health were significantly more numerous in the group with a high score), and being vaccinated against flu (those who did not regularly get vaccinated against influenza were significantly more numerous in the group with a low score). These results are in line with previous literature concerning general health literacy: scores of health literacy are higher in younger adults [34], health care professionals [35], and those vaccinated against flu [36].

Comparison with results from other studies is not possible because DVL has never been measured before.

### Strengths and Limitations

This study is the very first to develop and validate a standardized instrument for assessing general DVL in people. It responds to the urgent need for similar scales to tackle vaccine-related misinformation [37], especially in relation to the COVID-19 pandemic. Measuring the DVL of individuals consulting the internet for information on COVID-19–related vaccination could inform health institutions, communication experts, and health care providers to plan and implement strategies to overcome gaps in DVL and promote vaccination [38]. Furthermore, analyses performed in this study are robust and based on an in-depth knowledge of psychometrics techniques. In particular, the use of the bifactorial model is justified by the fact that it considers correlations between items based on the general factor and the relations between the general factor. Items are not limited by the group factors. This model is largely applied in cognitive and psychological sciences [39].

This study is not without limitations. Items were defined a priori based on existing scales but limited to 7. A larger number of items might have provided a more exhaustive coverage of DVL factors. The population under study was not representative of French adults given that it comprised a high number of women (2971/3738, 79.48%), students (3498/3783, 93.58%), and young people (29.2 years) [40], compared with the general population [41]. However, the sample was large enough to assess the relevance of the scale. Low ICC values in some separated items might be explained by an inaccurate phrasing. The ICCs of 3 items were low, which corresponds to a low reliability. Future instruments might be based on our scale, but we propose more precise wording according to the population of interest in a specific context (eg, cultural or sociodemographic characteristics).

### Conclusions

The DVL scale is the first instrument providing information on the way individuals understand, trust, and appraise vaccine-related information on the internet through 2 channels, namely, social media and government websites. The DVL scale has good psychometric properties in terms of content validity, dimensionality, and convergent and discriminant validity. Results show that the scale can be easily administered with well-grounded outcomes. It is a screening instrument contributing to detect people who need to be supported in navigating vaccine-related information online. It can be used in questionnaires to identify profiles of web users who could be influenced by anti-vax movements, for instance. Providing the instructions to look for online information and to understand its content is the key to spreading good vaccine-related information and promoting vaccination in general [42]. The scale can be used to measure DVL in the French population and translated validated versions could be proposed internationally.

## Appendix

Multimedia Appendix 1 Original items of the DVL scale (French). DVL scale: Digital Vaccine Literacy scale.

Multimedia Appendix 2 Comparison of responses to the 7 DVL items according to sociodemographic characteristics (n=848). DVL: digital vaccine literacy.

## Abbreviations

CFA: confirmatory factor analysis
CNIL: Commission Nationale de l'Informatique et des Libertés
COSMIN: Consensus-Based Standards for the Selection of Health Measurement Instruments
CPP: Comité de Protection des Personnes
DVL: digital vaccine literacy
EFA: exploratory factor analysis
EU GDPR: European Union General Data Protection Regulation
ICC: intraclass correlation coefficient

## Data Availability Statement

All data generated or analyzed during this study are included in this published article. The full data set is available upon request from the CONFINS cohort team.
