# Supplementary material for: Measuring Digital Vaccine Literacy: Development and Psychometric Assessment of the Digital Vaccine Literacy Scale
Source: J Med Internet Res. 2022 Dec 14;24(12):e39220. doi: 10.2196/39220 (PMC9798258; doi:10.2196/39220)
Supplement: Multimedia Appendix 1 [file jmir_v24i12e39220_app1.docx]

**MULTIMEDIA APPENDIX 1. ORIGINAL ITEMS OF THE DVL SCALE (French)**

**Concernant la recherche d’informations sur la vaccination, à quel point êtes-vous d’accord avec les propositions suivantes ?**

**Réponses :** Pas d’accord ; Plutôt pas d’accord ; Plutôt d’accord ; D’accord ; Je ne sais pas, je ne cherche pas d’informations sur la vaccination.

1. Je trouve que les informations concernant la vaccination sur les réseaux sociaux et les forums sont compréhensibles
2. Je trouve que les informations concernant la vaccination sur les sites gouvernementaux sont compréhensibles
3. Je suis capable de reconnaître les fake news sur le thème de la vaccination
4. J’ai confiance dans les informations fournies par les sites gouvernementaux
5. Je trouve que les informations sur la vaccination sur les réseaux sociaux sont valides
6. Lorsque je prends connaissance d'une information sur la vaccination en ligne, je croise plusieurs sources afin de vérifier sa validité
7. Je pense que les informations trouvées en ligne peuvent influencer mon choix de me faire vacciner
