# Supplementary material for: Measuring Digital Vaccine Literacy: Development and Psychometric Assessment of the Digital Vaccine Literacy Scale
Source: J Med Internet Res. 2022 Dec 14;24(12):e39220. doi: 10.2196/39220 (PMC9798258; doi:10.2196/39220)
Supplement: Multimedia Appendix 2 [file jmir_v24i12e39220_app2.docx]

**Multimedia Appendix 2.** Comparison of responses to the 7 DVL items according to sociodemographic characteristics (n=848). DVL: digital vaccine literacy.

| Item | | Gender (n=848) | | | Age (n=835) | | | Studying/working in the field of health (n=763) | | | Parent (n=848) | | | Regular vaccination against flu (n=848) | | |
| --- | --- | --- | --- | --- | --- | --- | --- | --- | --- | --- | --- | --- | --- | --- | --- | --- |
|  | | Men, n (%) | Women, n (%) | *P* value | No, n (%) | Yes, n (%) | *P* value | No, n (%) | Yes, n (%) | *P* value | No, n (%) | Yes, n (%) | *P* value | No, n (%) | Yes, n (%) | *P* value |
| **1** | |  |  | .11 |  |  | .003 |  |  | .88 |  |  | .05 |  |  | .58 |
|  | Disagree | 125 (54.8) | 301 (48.5) |  | 311 (47.6) | 109 (59.9) |  | 187 (51.1) | 205 (51.6) |  | 325 (48.5) | 101 (56.7) |  | 276 (49.6) | 150 (51.5) |  |
|  | Agree | 103 (45.2) | 319 (51.5) |  | 342 (52.4) | 73 (40.1) |  | 179 (48.9) | 192 (48.4) |  | 345 (51.5) | 77 (43.3) |  | 281 (50.4) | 141 (48.5) |  |
| **2** | |  |  | .12 |  |  | .41 |  |  | <.001 |  |  | .91 |  |  | <.001 |
|  | Disagree | 28 (12.3) | 103 (16.6) |  | 95 (14.5) | 31 (17.0) |  | 82 (22.4) | 36 (9.1) |  | 103 (15.4) | 28 (15.7) |  | 104 (18.7) | 27 (9.3) |  |
|  | Agree | 200 (87.7) | 517 (83.4) |  | 558 (85.5) | 151 (83.0) |  | 284 (77.6) | 361 (90.9) |  | 567 (84.6) | 150 (84.3) |  | 453 (81.3) | 264 (90.7) |  |
| **3** | |  |  | .003 |  |  | .48 |  |  | <.001 |  |  | .17 |  |  | <.001 |
|  | Disagree | 20 (8.8) | 118 (19.0) |  | 104 (15.9) | 33 (18.1) |  | 79 (21.6) | 36 (9.1) |  | 103 (15.4) | 35 (19.7) |  | 111 (19.9) | 27 (9.3) |  |
|  | Agree | 208 (91.2) | 502 (81.0) |  | 549 (84.1) | 149 (81.9) |  | 287 (78.4) | 361 (90.9) |  | 567 (84.6) | 143 (80.3) |  | 446 (80.1) | 264 (90.7) |  |
| **4** | |  |  | .008 |  |  | .16 |  |  | <.001 |  |  | .20 |  |  | <.001 |
|  | Disagree | 17 (7.5) | 88 (14.2) |  | 75 (11.5) | 28 (15.4) |  | 66 (18.0) | 21 (5.3) |  | 78 (11.6) | 27 (15.2) |  | 89 (16.0) | 16 (5.5) |  |
|  | Agree | 211 (92.5) | 532 (85.8) |  | 578 (88.5) | 154 (84.6) |  | 300 (82.0) | 376 (94.7) |  | 592 (88.4) | 151 (84.8) |  | 468 (84.0) | 275 (94.5) |  |
| **5** | |  |  | .89 |  |  | .43 |  |  | .12 |  |  | .18 |  |  | .89 |
|  | Disagree | 203 (89.0) | 550 (88.7) |  | 584 (89.4) | 159 (87.4) |  | 319 (87.2) | 360 (90.7) |  | 600 (89.6) | 153 (86.0) |  | 494 (88.7) | 259 (89.0) |  |
|  | Agree | 25 (11.0) | 70 (11.3) |  | 69 (10.6) | 23 (12.6) |  | 47 (12.8) | 37 (9.3) |  | 70 (10.4) | 25 (14.0) |  | 63 (11.3) | 32 (11.0) |  |
| **6** | |  |  | .42 |  |  | .44 |  |  | .94 |  |  | .20 |  |  | .54 |
|  | Disagree | 39 (17.1) | 92 (14.8) |  | 105 (16.1) | 25 (13.7) |  | 56 (15.3) | 60 (15.1) |  | 109 (16.3) | 22 (12.4) |  | 83 (14.9) | 48 (16.5) |  |
|  | Agree | 189 (82.9) | 528 (85.2) |  | 548 (83.9) | 157 (86.3) |  | 310 (84.7) | 337 (84.9) |  | 561 (83.7) | 156 (87.6) |  | 474 (85.1) | 243 (83.5) |  |
| **7** | |  |  | .03 |  |  | .93 |  |  | <.001 |  |  | .78 |  |  | <.001 |
|  | Disagree | 119 (52.2) | 270 (43.5) |  | 299 (45.8) | 84 (46.2) |  | 145 (39.6) | 209 (52.6) |  | 309 (46.1) | 80 (44.9) |  | 222 (39.9) | 167 (57.4) |  |
|  | Agree | 109 (47.8) | 350 (56.5) |  | 354 (54.2) | 98 (53.8) |  | 221 (60.4) | 188 (47.4) |  | 361 (53.9) | 98 (55.1) |  | 335 (60.1) | 124 (42.6) |  |
